# BMS-794833 reduces anlotinib resistance in osteosarcoma by targeting the VEGFR/Ras/CDK2 pathway

**DOI:** 10.1016/j.jbo.2024.100594

**Published:** 2024-03-16

**Authors:** Qingtao Meng, Jian Han, Peng Wang, Chenxu Jia, Mingyang Guan, Bolun Zhang, Wenzhi Zhao

**Affiliations:** a Department of Orthopedics, The Second Affiliated Hospital of Dalian Medical University, Dalian 116028, China; b Department of Orthopedics, Dalian NO.3 People’s Hospital, Dalian 116091, China; c Department of Orthopedic Surgery, The First Affiliated Hospital of Shandong First Medical University, Jinan, China

**Keywords:** Osteosarcoma, Anlotinib, VEGFR, CDK2, Synergy effect

## Abstract

**Background:**

Osteosarcoma, a tumor that originates from bone cells, has a poor prognosis and a high degree of malignancy. Anlotinib, a small-molecule multi-target tyrosine kinase inhibitor (TKI), is the first-line drug in treating osteosarcoma, especially in late-stage osteosarcoma. However, patients often develop resistance after using anlotinib for a certain period, which poses a challenge to its further clinical application. Recently, several TKIs, for instance regorafenib and cabozantinib, have showed clinical interest in treating osteosarcoma and target both vascular endothelial growth factor receptor (VEGFR) and mesenchymal epithelial transition factor (c-MET). Therefore, the identification of new TKI warrants further investigation.

**Methods:**

We performed CCK8 aasays to confirm that BMS-794833 sensitization osteosarcoma cells to anlotinib. Bioinformatics analysis and rescue experiments showed that the reduce of resistance were dependent on the VEGFR/Ras/CDK2 pathway. Cell line based xenograft model were used to demonstrate that BMS-794833 and anlotinib could synergistically treat OS.

**Results:**

Here, we found that BMS-794833 reduced anlotinib resistance in osteosarcoma by targeting the VEGFR/Ras/CDK2 pathway. CCK8 assay showed that BMS-794833 significantly improved the resistance of osteosarcoma cells to anlotinib. The results of rescue experiments showed that the regulatory effects of BMS-794833 on the proliferation and drug resistance of osteosarcoma cells were dependent on the VEGFR/Ras/CDK2 pathway. In addition, BMS-794833 affected the resistance of osteosarcoma cells to anlotinib through epithelial–mesenchymal transition (EMT) and apoptosis pathways. More importantly, BMS-794833 and anlotinib exerted synergistic therapeutic effects against osteosarcoma *in vivo*.

**Conclusion:**

Altogether, this study reveals a new (VEGFR)-targeting drug that can be combined with anlotinib for the treatment of osteosarcoma, which provides an important theoretical basis for overcoming anlotinib resistance.

## Highlights

• BMS-794833 reduced anlotinib resistance in osteosarcoma.
• BMS-794833 regulates the resistance were dependent on the VEGFR/Ras/CDK2 pathway.
• BMS-794833 affected the resistance through epithelial–mesenchymal transition (EMT) and apoptosis pathways.
• BMS-794833 and anlotinib exerted synergistic therapeutic effects against osteosarcoma.

## 1 Introduction

Osteosarcoma is a predominant primary malignant bone cancer that is most commonly observed in teenagers and young adults [1]. It has a poor prognosis and a high degree of malignancy [2]. Surgery combined with postoperative adjuvant drug treatment is the primary method for early-stage osteosarcoma [3]. Targeted therapy is directed against specific molecules or pathways that are crucial for the growth and progression of cancer [4]. Because the early symptoms of osteosarcoma are relatively insidious, some patients are diagnosed at an advanced stage and early lung metastasis is common [5]. Targeted therapy for advanced osteosarcoma focuses on specific molecules aiding tumor growth and progression [6]. Unlike traditional chemotherapy, which impacts all rapidly dividing cells, targeted therapy selectively attacks cancer cells, reducing the risk of side effects [7]. However, not all osteosarcomas possess the molecular markers required for effective targeted therapy [6]. Moreover, resistance to targeted agents may develop, rendering treatments ineffective over time [8]. Therefore, it is necessary to develop novel strategies for improving the sensitivity of osteosarcoma to targeted drugs.

Tyrosine kinase inhibitors (TKIs) are drugs designed to block TKs that are crucial for various cellular processes such as cell growth, division, and differentiation [9], [10]. TKIs exert anti-tumor effects by attenuating angiogenesis, cell proliferation, and cell survival by inhibiting aberrant TK signaling, especially in cancers with mutations or overexpression of TKs [11]. Although TKIs hold promise in the treatment of osteosarcoma, their efficacy is currently under investigation and challenges such as the development of resistance persist [12]. Anlotinib is a multi-target TKI with pronounced anti-tumor effects owing to its ability to inhibit angiogenesis and tumor cell proliferation [13]. It specifically targets VEGFR, platelet-derived growth factor receptor (PDGFR), and fibroblast growth factor receptor (FGFR) to block pathways associated with tumor growth and metastasis [14]. It exerts anti-tumor effects by suppressing angiogenesis, thereby disrupting the supply of nutrients to the tumor, and influences various stages of tumor cell proliferation and survival to inhibit tumor growth and metastasis [15]. It has demonstrated therapeutic efficacy in multiple malignancies, including lung cancer and soft tissue sarcoma, by delaying tumor progression and improving patient survival [16]. In early-stage and middle-stage osteosarcoma, anlotinib inhibits the growth and metastasis of primary tumors by disrupting angiogenesis and hindering the supply of essential nutrients and oxygen to tumor cells [17]. In late-stage osteosarcoma, especially when conventional treatments fail or are not viable, anlotinib offers a palliative option to manage the disease and improve the quality of life by controlling tumor growth and potentially alleviating some disease-related symptoms [18]. However, the development of drug resistance and the genetic heterogeneity of osteosarcoma may limit the effectiveness and applicability of anlotinib [6], [19]. Combining anlotinib with other VEGFR-targeting drugs, such as sorafenib, is a potential approach to amplifying its therapeutic effects against osteosarcoma [20], [21]. In addition, obstructing multiple tumor angiogenesis-associated pathways simultaneously may synergistically suppress tumor growth [22]. However, these combinatorial approaches have certain limitations. To date, most combination strategies have been verified primarily in cell cultures or animal models, with only a few strategies demonstrating the potential for clinical translation. Moreover, combining drugs may increase the risk of side effects. At present, studies are ongoing to identify novel, more efficient drugs targeting VEGFR to improve the sensitivity of osteosarcoma to anlotinib [23].

BMS-794833 is a potent and specific inhibitor of both VEGFR (vascular endothelial growth factor receptor) and MET (mesenchymal epithelial transition factor) [24]. These receptors play an essential role in tumor angiogenesis, invasion, and metastasis and hence are promising targets for therapeutic intervention [25]. BMS-794833 has shown potential as an anti-cancer agent in preclinical studies and phase I clinical trials [26]. It has been reported to suppress tumor growth in mice with breast cancer and attenuate the pro-tumoral properties of tumor-associated macrophages (TAMs) *in vitro* [24]. BMS-794833 is a multitargeted compound that inhibits c-Met, VEGFR2, Ron, Axl, and Flt3 [24] and exerts dose-dependent therapeutic effects against multiple tumors [27]. It targets VEGFR and MET simultaneously, exhibiting a dual mechanism of action. Therefore, it is considered a promising therapeutic agent for cancers, especially those resistant to single-target therapies [26]. However, the specific effects and mechanism of action of BMS-794833 in osteosarcoma have not been reported to date.

This study demonstrated that BMS-794833 mitigated anlotinib resistance in osteosarcoma by acting on the VEGFR/Ras/CDK2 pathway. Notably, BMS-794833 augmented the sensitivity of osteosarcoma cells to anlotinib *in vitro*. The effects of BMS-794833 on the proliferation and drug resistance of osteosarcoma cells were found to be dependent on the VEGFR/Ras/CDK2 signaling pathway. Concurrently, BMS-794833 modulated anlotinib resistance through the EMT and apoptosis pathways. Combination treatment with BMS-794833 and anlotinib exhibited synergistic anti-cancer activity in both *in vivo* and *in vitro* models of osteosarcoma.

## 2 Methods and materials

### 2.1 Cell culture

The human osteosarcoma cell lines U2OS, MG63, hFOB1.19, SaOS-2 and the human embryonic kidney cell line HEK-293 T were obtained from the National Certified Cell Culture Collection Center (Shanghai, China). The U2OS and SaOS-2 cells were cultured in McCoy's 5a Medium (Invitrogen) supplemented with 10 % fetal bovine serum and 1 % antibiotics. The MG63, hFOB1.19 and HEK-293 T cells were cultured in Dulbecco’s Modified Eagle Medium (DMEM; Gibco) supplemented with 10 % fetal bovine serum and 1 % antibiotics.

### 2.2 Transfection

HEK-293 T cells were seeded in 6-well plates, and psPAX2 and pMD2.G plasmids were co-transfected into the cells after 24 h. The viral solution was collected and filtered using a 0.45-μm membrane filter. Subsequently, osteosarcoma cells were infected with the viral solution and screened with puromycin for 2 weeks to establish stably transfected cell lines.

### 2.3 Protein extraction and western blotting

Cells were lysed with a protein extraction buffer to disrupt and dissolve proteins. The cell lysates were centrifuged to remove debris and organelles, and supernatants containing purified proteins were collected. The concentration of proteins in the supernatants was determined via BCA assay. The extracted proteins were separated on polyacrylamide gels based on their molecular weight and transferred to a PVDF membrane. The membrane was treated with 5 % skimmed milk to block non-specific binding sites and incubated with primary antibodies against target proteins. Subsequently, the membrane was incubated with corresponding secondary antibodies, and protein bands were visualized via enhanced chemiluminescence.

### 2.4 RNA extraction and qRT-PCR

Cells were lysed using an RNA extraction kit (Invitrogen, USA). After the cell lysates were centrifuged to remove debris and contaminants such as proteins, supernatants with purified RNA were collected. The concentration and purity of the extracted RNA were assessed using a spectrophotometer. Subsequently, the RNA was used as a template for synthesizing cDNA using a reverse transcription kit. The resulting cDNA was used as a template for PCR, in which specific primers were used to amplify the DNA sequence of the target gene. The primer sequences used as follows:

CDK2-F: CCAGGAGTTACTTCTATGCCTGA

CDK2-R: TTCATCCAGGGGAGGTACAAC

GAPDH-F: GTCTCCTCTGACTTCAACAGCG

GAPDH-R: ACCACCCTGTTGCTGTAGCCAA

### 2.5 CCK8 assay

Osteosarcoma cells U2OS and MG63 were inoculated in 96-well plates at a density of 6 × 10^3^ cells per well and incubated at 37℃ and 5 % CO_2_ for 24 h. After the cells were completely attached to the wall, different drugs were added and incubated for 24 h according to the experimental design. To detect the IC50 value of Anlotinib, we used different concentrations of DMSO, BMS-794833, hVEGF-IN-1 and PD173074 to treat cells. Besides, for measuring the cell proliferation capacity of osteosarcoma, different concentrations of BMS-794833 (10uM and 20uM) were added to cells to detect the effects of the BMS-794833 on osteosarcoma cells. Finally, the cells were incubated with CCK-8 reagent for 1 h. The absorbance of the products produced in the culture was measured using an enzyme marker, and the cell survival rate was calculated. The IC50 value was evaluated using a curve fitting method.

### 2.6 Colony formation assay

Osteosarcoma cells were seeded in 6-well plates at a density of 0.8 × 10^3^ cells per well and incubated at 37 °C and 5 % CO_2_ for 10 days. Subsequently, the cells were washed with PBS and stained with crystal violet. Cell colonies were observed and imaged using an inverted microscope (IX53, Olympus, Japan).

### 2.7 Apoptosis assay

Drug-treated osteosarcoma cells were collected to obtain a homogeneous single-cell suspension. After the cells were washed, centrifuged, and resuspended, apoptosis was assessed using a flow cytometer. In addition, the percentages and numbers of distinct cell subsets were calculated and recorded.

### 2.8 Transwell migration assay

To investigate the the drug effects of BMS-794833 on the cell migration of osteosarcoma cells, U2OS and MG63 cells treated with different concentrations of BMS-794833 (10uM and 20uM) were added to the upper transwell chamber. A serum-free medium was added to the upper transwell chamber, whereas a medium containing 10 % fetal bovine serum and drugs at different concentrations were added to the lower chamber. The cells were incubated for 48 h, fixed, and stained with crystal violet. Subsequently, the number of cells that migrated to the lower chamber was counted using a microscope.

### 2.9 Mice experiments

Male nude BALB/C mice (age, 4 weeks) were purchased from Liaoning Changsheng Biological Co., LTD and housed in a specific pathogen-free (SPF) environment. Animal experiments were approved by the Ethics Committee of Dalian Third Hospital and performed in accordance with established guidelines (2022–137-002). To establish tumor xenograft models, the mice were subcutaneously injected with osteosarcoma cells (1x10^6^ U2OS cells in 100 μl PBS). The deep anesthesia was used to control and treat pain. After transplantation, tumor volume was measured every 4 days. When the tumor volume reached 50 mm^3^, the mice were randomly divided into 4 groups, with 5 mice in each group, as follows: normal control (DMSO), BMS-794833, anlotinib, and BMS-794833 combined with anlotinib groups. The drugs were administered orally for 28 days (BMS-794833, 10 mg/kg in corn oil; anlotinib, 5 mg/kg in corn oil). The weight of mice was recorded 48 h after injection. Mice were monitored daily and euthanized when they became moribund.

### 2.10 Immunohistochemical experiments

Mouse tumor tissues were collected, fixed in 4 % paraformaldehyde, embedded in paraffin, and cut into 4-µm-thick sections. The tissue sections were dewaxed in xylene and dehydrated in a graded series of alcohol. The sections were treated with 3 % hydrogen peroxide and heated for antigen retrieval and blocked with 5 % BSA. Subsequently, the sections were incubated with primary antibody (Ki67, 1:100; Cell Signaling Technology) and corresponding secondary antibody, stained with DAB, and counter-stained with hematoxylin. Randomly select 5 200x visual fields and calculate the proportion of positive tumor cells in all tumor cells. This judgment process should be conducted by two profcient pathologists independently.

### 2.11 Data collection

RNA-seq data were extracted from an osteosarcoma dataset in the GEO database and TCGA database.

### 2.12 Differential expression of genes

The limma package in R was used to screen for differentially expressed genes between osteosarcoma and normal samples, with the screening threshold being set to *P*-values of < 0.05 and logFC values of > 0.5. Volcano maps and heat maps were generated to visualize the results.

### 2.13 Batch univariate Cox and Kaplan–Meier survival analysis

Batch univariate Cox analysis involves conducting separate univariate analyses on multiple predictor variables within the same Cox proportional hazards model, yielding hazard ratios and significance values for each variable. Furthermore, Kaplan–Meier (KM) analysis was used to compare patient survival among groups. Survival curves were plotted for distinct groups and compared using the log-rank test.

### 2.14 Gene set enrichment analysis

Gene set enrichment analysis (GSEA) is a common method used to assess the enrichment patterns of gene sets based on gene expression data. Genes in a dataset are ranked based on their expression levels, and an enrichment score is calculated to evaluate their enrichment patterns. The enrichment score reflects the cumulative deviation of the gene set within the ranked gene list, which enables the assessment of whether the gene set is enriched at specific positions. Enrichment plots were generated to visualize the results.

### 2.15 DAVID enrichment analysis

DAVID is a widely used bioinformatic tool for conducting functional enrichment analysis of genes or proteins. Lists of differentially expressed genes were submitted to DAVID, and the appropriate species was selected for functional annotation of the submitted gene list, associating genes with specific biological processes, molecular functions, and cellular components.

### 2.16 Statistical analysis

Statistical analysis was performed using the GraphPad Prism 8.0 software. Data were expressed as the mean ± standard deviation. Student’s *t*-test was used to analyze differences between groups. A *P*-value of < 0.05 was considered statistically significant.

## 3 Results

### 3.1 BMS-794833 enhanced the sensitivity of osteosarcoma cells to anlotinib

**Fig. 1:**
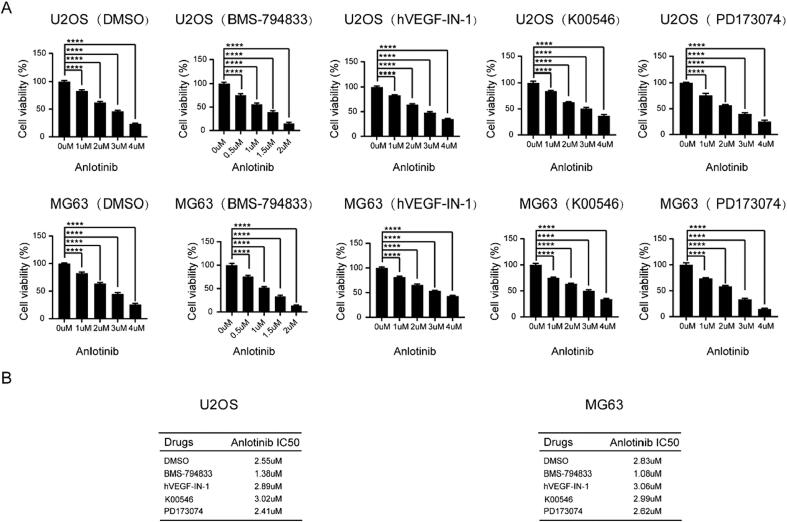
BMS-794833 could enhance the sensitivity of osteosarcoma to Anlotinib. (A-B) The U2OS and MG63 cells were treated with DMSO, BMS-794833, hVEGF-IN-1, K00546 and PD173074 respectively, and the viability of the cells was detected by CCK8 assay, and the IC50 value was calculated. All experiments carried out in triplicate. *P < 0.05 and **P < 0.01 comparing with control group with t-Test in GraphPad Prism 8.0 software.

Given that the combined use of multitargeted drugs may enhance their sensitivity, we attempted to identify novel small molecules that can overcome anlotinib resistance by targeting VEGFR, PDGFR, and FGFR. Molecular docking revealed four candidate drugs that potentially target VEGFR, PDGFR, and FGFR, including BMS-794833, hVEGF-IN-1, K00546, and PD173074. Osteosarcoma cells were treated with these four drugs in combination with anlotinib, and CCK8 assay was used to measure the IC50 value of anlotinib. Remarkably, the combined use of anlotinib and BMS-794833 decreased the IC50 value of anlotinib (Fig. 1A-B). These findings indicated that BMS-794833 enhanced the sensitivity of osteosarcoma cells to anlotinib.

### 3.2 BMS-794833 inhibited the proliferation of osteosarcoma cells

**Fig. 2:**
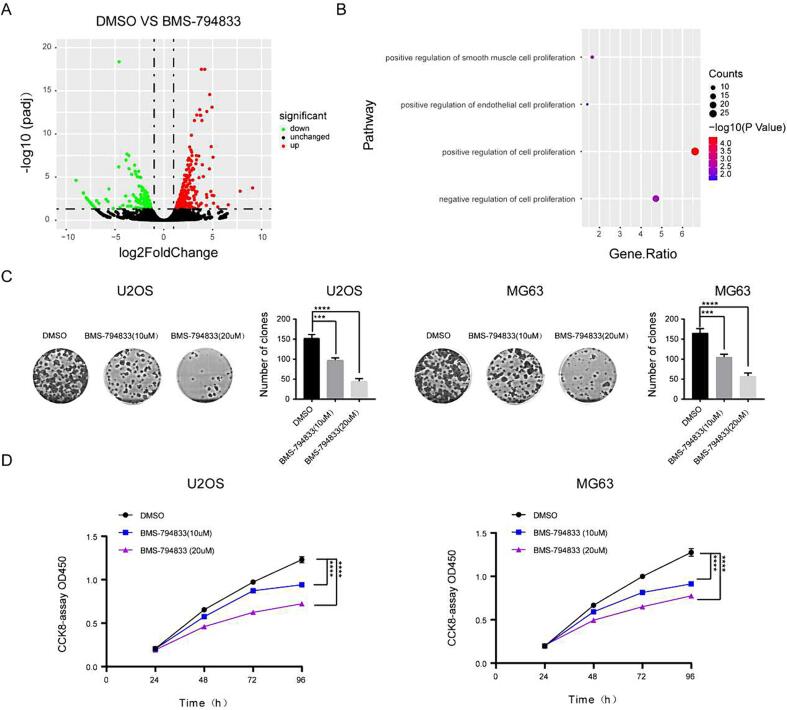
BMS-794833 inhibits the proliferation of osteosarcoma cells. (A) The volcano map showed the differential genes between DMSO treated group and BMS-794833 treated group. (B) A bubble plot of GSEA enrichment analysis. (C-D) U2OS and MG63 cells were treated with BMS-794833 at the shown concentrations (10 μM, 20 μM) for 24 h, and the proliferation of the cells was detected by clonal formation and CCK8 assay. In the control group, cells were treated with DMSO for the same time. All experiments carried out in triplicate. *P < 0.05 and **P < 0.01 comparing with control group with t-Test in GraphPad Prism 8.0 software.

To clarify the role of BMS-794833 in osteosarcoma, we examined the IC50 of BMS794833-treated osteosarcoma cells and normal cells by CCK8 assay. The results showed that BMS794833-treated osteosarcoma cells had a lower IC50 than normal cells. These results indicated that BMS794833 has anticancer effect in osteosarcoma cells (Fig. S1A-B). To examine the effects of BMS-794833 on the malignant progression of osteosarcoma and elucidate the underlying mechanisms, MG63 cells were treated with BMS-794833 or DMSO respectively and subjected to RNA-seq. Differential expression analysis showed that the expression of 1864 genes was significantly altered after BMS-794833 treatment (948 upregulated and 916 downregulated genes) (Fig. 2A). Subsequently, DAVID was used for enrichment analysis of the differentially expressed genes. The results showed that the genes were significantly enriched in proliferation-related pathways (Fig. 2B and Fig. S2). To validate the impact of BMS-794833 on the proliferation of osteosarcoma, osteosarcoma cells were treated with BMS-794833 at increasing concentrations. The results of CCK8 and colony formation assays showed that BMS-794833 suppressed the proliferation of osteosarcoma cells in a concentration-dependent manner (Fig. 2C, D).

### 3.3 BMS-794833 inhibited the proliferation of osteosarcoma cells through the VEGFR/Ras/CDK2 axis

**Fig. 3:**
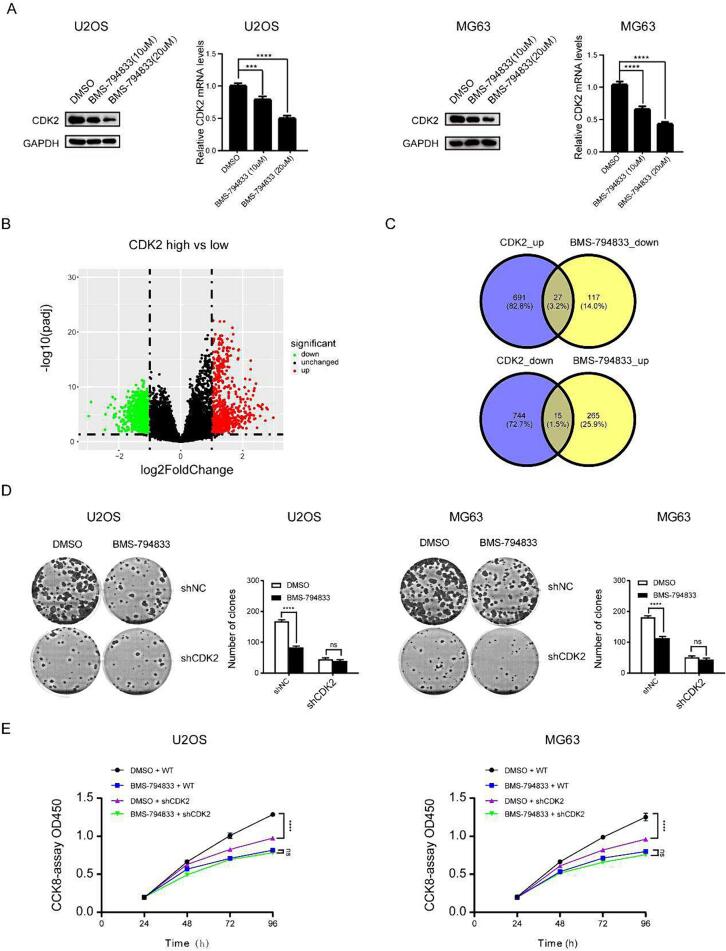
BMS-794833 inhibits osteosarcoma cell proliferation through the VEGFR/Ras/CDK2 axis. (A) U2OS and MG63 cells were treated with BMS-794833 at the shown concentrations (10 μM, 20 μM) for 24 h, and the expression levels of CDK2 protein and nucleic acid were detected by western blot assay and RT-PCR. (B) The volcano map showed the differential genes of the high and low expression CDK2 groups. (C) Venn diagram shows overlapping genes. DMS-794833 and DMSO were used to treat U2OS and MG63 cells transfected with shNC or shCDK2, and cell proliferation was measured by colony formation (D) and CCK8 (E). All experiments carried out in triplicate. *P < 0.05 and **P < 0.01 comparing with control group with t-Test in GraphPad Prism 8.0 software.

The abovementioned results indicated that BMS-794833 inhibited the proliferation of osteosarcoma cells; however, whether its mechanism of action was consistent with the prediction made based on molecular docking data was unclear. Pathway enrichment analysis showed that the differentially expressed genes induced by BMS-794833 were enriched in the Ras pathway (Fig. S3A). The Ras pathway is a crucial component of the VEGFR signaling pathway, which is consistent with our previous expectations. Because CDK2 is an important gene in the Ras pathway, we examined its expression in cells treated with BMS-794833 at increasing concentrations. The results showed that treatment with BMS-794833 significantly decreased the mRNA and protein expression of CDK2 in a concentration-dependent manner (Fig. 3A), indicating that BMS-794833 could regulate the VEGFR/Ras/CDK2 axis. Based on the expression pattern of CDK2 in osteosarcoma samples in the GEO dataset and the relationship of CDK2 with survival in patients with osteosarcoma in TCGA dataset, CDK2 was found to play an important role in the development of osteosarcoma. However, whether BMS-794833 regulated osteosarcoma through the VEGFR/Ras/CDK2 axis was unclear. To validate this hypothesis, osteosarcoma samples in TCGA dataset were divided into high- and low-CDK2 expression groups based on the median expression of CDK2. A total of 1435 differentially expressed genes (691 upregulated and 744 downregulated genes) were identified between the two groups (Fig. 3B). The upregulated genes were intersected with downregulated genes induced by BMS-794833 to obtain a set of genes. Similarly, the downregulated genes were intersected with upregulated genes induced by BMS-794833, resulting in 42 regulated genes (Fig. 3C). These results suggest that the regulatory effects of BMS-794833 on osteosarcoma depend on CDK2. Rescue experiments were performed to verify these results. Colony formation and CCK8 assays revealed that BMS-794833 inhibited the proliferation of osteosarcoma cells in the shNC group; however, these inhibitory effects were not significant in the sh-CDK2 group (Fig. 3D-E). Altogether, these results indicate that BMS-794833 inhibits the proliferation of osteosarcoma cells through the VEGFR/Ras/CDK2 axis and enhances the sensitivity of osteosarcoma cells to anlotinib.

### 3.4 BMS-794833 affected the resistance of osteosarcoma to anlotinib through the EMT and apoptosis pathways

**Fig. 4:**
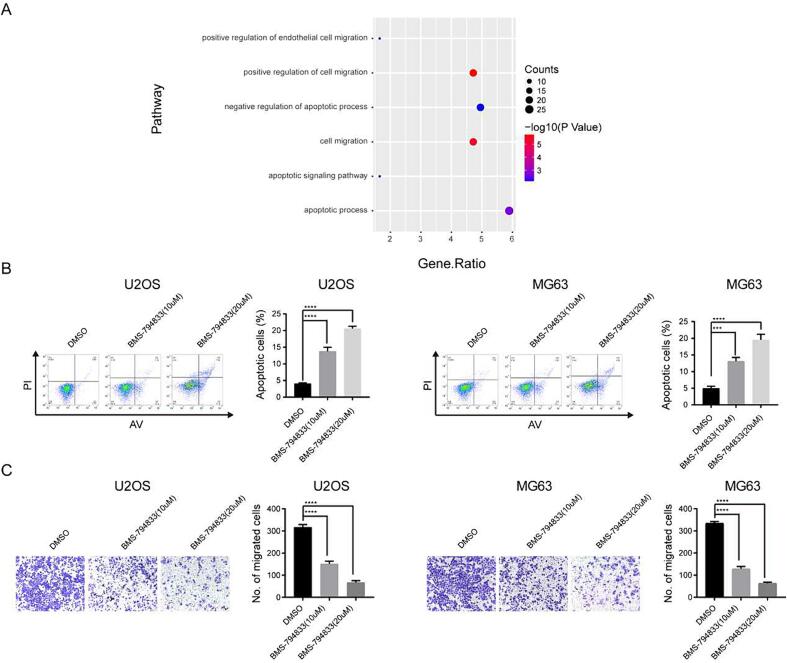
BMS-794833 may affect the resistance of osteosarcoma to anlotinib through EMT and apoptosis pathways. (A) GSEA pathway enrichment map. (B) U2OS and MG63 cells were treated with BMS-794833 containing the shown concentrations (10 μM, 20 μM) for 24 h, and apoptosis was detected by flow cytometry. (C) Cell migration was detected by transwell assay. In the control group, cells were treated with DMSO for the same time. All experiments carried out in triplicate. *P < 0.05 and **P < 0.01 comparing with control group with t-Test in GraphPad Prism 8.0 software.

According to the results of differential expression and enrichment analyses based on RNA-seq data, the differentially expressed genes induced by BMS-794833 were found to be significantly enriched in the EMT and apoptosis pathways (Fig. 4A, Fig. S4). Previous studies have demonstrated that EMT alters the gene expression patterns of tumor cells, leading to the development of chemotherapy resistance. In addition, apoptosis induces a series of intracellular signaling cascades to promote tumor cell death, thereby contributing to drug resistance. Therefore, we hypothesized that the regulatory effects of BMS-794833 on anlotinib resistance in osteosarcoma were associated with EMT and apoptosis. To validate this hypothesis, flow cytometry was performed on osteosarcoma cells treated with BMS-794833 at increasing concentrations. The results showed that treatment with BMS-794833 significantly increased the proportion of apoptotic cells in a concentration-dependent manner (Fig. 4B). Additionally, transwell assay indicated that BMS-794833 significantly attenuated the migratory ability of osteosarcoma cells in a concentration-dependent manner (Fig. 4C). These results suggest that BMS-794833 affects anlotinib resistance in osteosarcoma through the EMT and apoptosis pathways.

### 3.5 BMS-794833 and anlotinib synergistically inhibited the malignant progression of osteosarcoma *in vitro*

**Fig. 5:**
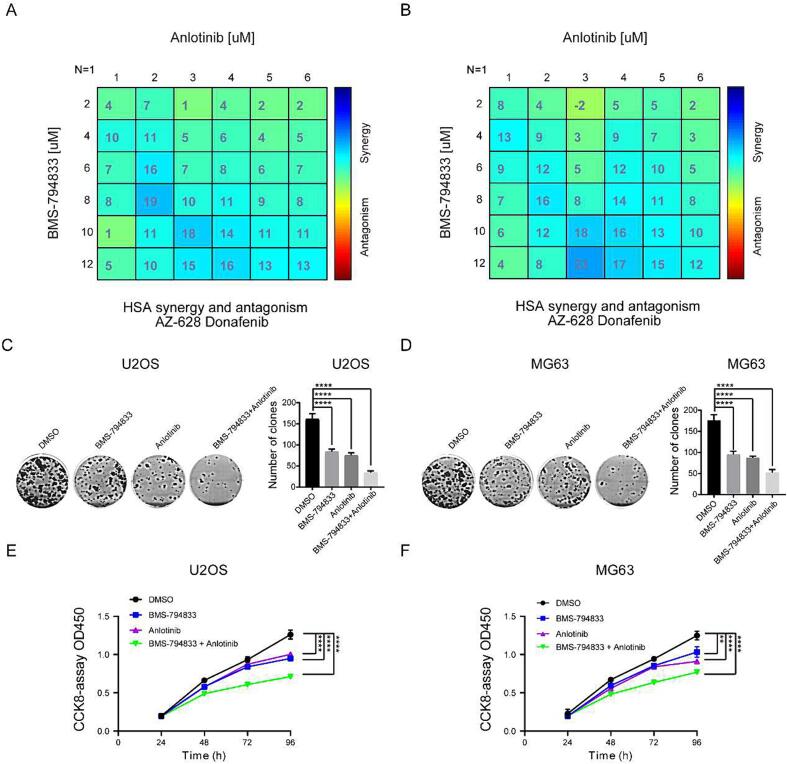
BMS-794833 and Anlotinib synergistically inhibit the malignant progression of osteosarcoma cells *in vitro*. (A-B) 2D visualization of synergy between BMS-794833 and anlotinib at various concentrations in two different osteosarcoma cell cultures (24 h treatment) by combenefit2. (C-D) Clonal formation assay of MG63 and U2OS cells treated with BMS-794833 and anlotinib, either alone or in combination. (E-F) CCK8 assay of MG63 and U2OS cells treated with BMS-794833 and anlotinib, either alone or in combination. All experiments carried out in triplicate. *P < 0.05 and **P < 0.01 comparing with control group with t-Test in GraphPad Prism 8.0 software.

Anlotinib is a well-known small-molecule inhibitor targeting the VEGFR pathway. As a monotherapy, anlotinib has limited inhibitory effects on tumor development. The abovementioned results suggest that BMS-794833 inhibits the proliferation of osteosarcoma cells through the VEGFR/Ras/CDK2 axis. However, whether the combined use of BMS-794833 and anlotinib exerts synergistic anti-tumor effects remains unclear. Therefore, we treated osteosarcoma cells with a combination of anlotinib and BMS-794833. The results showed that the combined use of the two drugs exerted significant synergistic effects against osteosarcoma cells (Fig. 5A-B and Fig. S5A-D). Furthermore, colony formation and CCK-8 assays revealed that the combined use of the two drugs significantly inhibited the proliferation of osteosarcoma cells (Fig. 5C-F). Altogether, these results indicated that BMS-794833 and anlotinib synergistically inhibited the malignant progression of osteosarcoma *in vitro*.

### 3.6 BMS-794833 and anlotinib synergistically inhibited the malignant progression of osteosarcoma *in vivo*

**Fig. 6:**
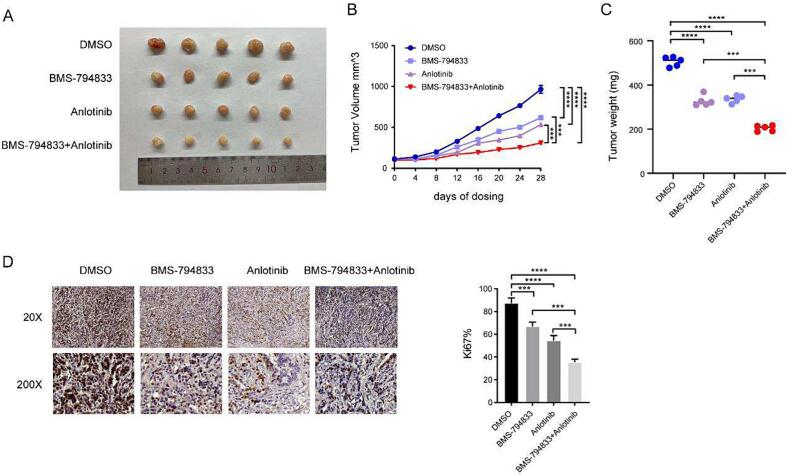
BMS-794833 and Anlotinib synergistically inhibit the malignant progression of osteosarcoma cells *in vivo*. Mice were treated with DMSO, anlotinib, BMS-794833, and a combination of anlotinib and BMS-794833. (A) Visible tumor formation and photographs of representative tumors removed from mice after treatment. Tumor volume (B) and tumor weight (C) changes in mice were examined every 4 days for 28 days during treatment. (D) The expression of Ki67 in xenografted tumor tissues of nude mice was observed by immunohistochemical staining (20×, 200 × ). Data are expressed as the mean ± SD of three independent experiments.

To verify the synergistic effects of BMS-794833 and anlotinib, U2OS cells were injected into nude mice to establish subcutaneous tumor xenograft models. A total of 20 nude mice were randomly divided into four groups as follows: control group, BMS-794833, anlotinib, and combination therapy groups. All mice survived during the treatment period. It is noteworthy that tumor volume and weight were lower in the BMS-794833 and anlotinib groups than in the control group, with the combination therapy group exhibiting the most significant inhibition of tumor growth (Fig. 6A-C). After 4 weeks of drug treatment, tumor tissues were harvested from mice and subjected to immunohistochemical analysis. As shown in Fig. 6D, the percentage of Ki67-positive proliferating tumor cells was lower in the BMS-794833 and anlotinib groups than in the control group, with the combination therapy group exhibiting the lowest proportion of Ki67-positive cells. These results indicated that BMS-794833 and anlotinib synergistically inhibited the growth of osteosarcoma *in vivo*. The mechanism diagram of this study is shown in Supplementary Fig. 6.

## 4 Discussion

Osteosarcoma has a poor prognosis and a high degree of malignancy. It is mostly diagnosed in the early and middle stages. Surgery plus postoperative adjuvant drug therapy is the primary treatment method for early- and middle-stage osteosarcoma. Anlotinib is a new oral RTK inhibitor that targets VEGFR-2, VEGFR-3, FGFR1-4, PDGFR-α, PDGFR-β, c-Kit, and Ret to suppress tumor growth and angiogenesis [18], [28], [29], [30], [31]. The VEGFR pathway plays an important role in osteosarcoma. Anlotinib, which mainly targets the VEGFR pathway, is an efficient drug for the targeted treatment of osteosarcoma. However, its widespread clinical application is challenging owing to the development of resistance after long-term use. In this study, we found that BMS-794833, a novel small-molecule drug, improved the sensitivity of osteosarcoma to anlotinib by targeting the VEGFR/Ras/CDK2 pathway. Altogether, this study proposes candidate drugs to overcome anlotinib resistance in osteosarcoma.

Drug resistance is a major challenge to successful treatment [32]. The mechanisms underlying drug resistance encompass altered drug uptake or efflux, drug target modifications, activation of alternative pathways, and enhanced DNA damage repair [33]. A notable molecular phenotypic change associated with drug resistance is epithelial–mesenchymal transition (EMT), in which epithelial cells acquire mesenchymal characteristics with enhanced migratory and invasive abilities and resistance to apoptosis [34]. Resistance to apoptosis is a characteristic of cancer and an important mechanism underlying drug resistance, in which tumor cells evade the killing effects of drugs and continue to proliferate and survive [35]. Understanding the mechanisms underlying drug resistance, from molecular shifts such as EMT to evasion of apoptosis, is important for developing more effective and durable therapeutic strategies [36]. This study showed that BMS-794833 enhanced the sensitivity of osteosarcoma cells to anlotinib by regulating the EMT and apoptosis pathways. The RNA-sequencing of anlotinib resistant osteosarcoma cell ine can better reflect the mechanism of the resistance to anlotinib. Thus, we will validate this finding by constructing resistant strains in future studies.

The VEGFR pathway plays an essential role in angiogenesis, which refers to the formation of new blood vessels from pre-existing ones [37]. When VEGFR is activated, it stimulates the Ras signaling cascade, leading to intracellular events that promote cell proliferation and angiogenesis [38]. In tumors, mutations or aberrant signaling of Ras are common and can lead to uncontrolled cell growth and resistance to apoptosis [39]. The continuous activation of Ras, often owing to mutations, can cause persistent stimulation of pathways that promote tumorigenesis [40]. Ras mutations or dysregulated Ras signaling have been associated with tumor progression and poor clinical outcomes in osteosarcoma. The important role of Ras in osteosarcoma emphasizes its potential as a therapeutic target, as disrupting Ras signaling can inhibit tumor growth and improve treatment responses [7]. CDK2 plays a crucial role in the Ras signaling cascade and in regulating cell cycle progression. The interaction between CDK2 and cyclins, especially cyclins E and A, drives the transition of cells from the G1 to the S phase, ensuring proper DNA replication [41]. Upon Ras activation, multiple downstream effectors get stimulated within the Ras pathway, eventually leading to the activation of CDK2. Activated CDK2 facilitates cell cycle progression, thereby promoting cell proliferation [42]. However, dysregulation or overactivity of CDK2 can be detrimental in the context of tumors. Unchecked CDK2 activity can lead to uncontrolled cell division, a hallmark of cancer. Mutations or abnormalities in components regulating CDK2, or in its upstream activators such as Ras, can sustain the active state of CDK2, promoting tumor growth and progression. CDK2 is considered to play an important role in osteosarcoma, as its overexpression or enhanced activity has been associated with aggressive tumor behavior, metastasis, and poor patient prognosis. Owing to its important role in tumor progression, CDK2 is a potential therapeutic target for osteosarcoma. Inhibition of CDK2 can suppress tumor growth and potentially enhance the efficacy of existing treatments. This study demonstrated that BMS-794833 regulated the proliferation of osteosarcoma cells through CDK2. Mechanistically, BMS-794833 enhanced the sensitivity of osteosarcoma to anlotinib by targeting the VEGFR/Ras/CDK2 pathway. These results provide a theoretical basis for combination therapy with BMS-794833 and anlotinib in osteosarcoma.

Anlotinib is a multi-targeted TKI that primarily inhibits angiogenesis and tumor cell proliferation. It has been shown to delay tumor progression and prolong patient survival in non-small cell lung cancer. In addition, it targets angiogenesis-related pathways, such as the VEGFR pathway, to inhibit primary tumor growth and metastasis in osteosarcoma and can be used in palliative care in advanced stages. Despite the substantial therapeutic potential of anlotinib, its effectiveness varies owing to the genetic heterogeneity of osteosarcoma. Additionally, patients may develop resistance over time, rendering the drug less effective. In most cases, the efficacy and sensitivity of anlotinib are improved by combining it with other drugs, especially those targeting the VEGFR pathway. BMS-794833 is a potent inhibitor that targets both VEGFR and MET and plays a crucial role in tumor angiogenesis and metastasis. Concerning the VEGFR pathway, BMS-794833 impedes angiogenesis, potentially limiting tumor growth by hindering the supply of necessary nutrients and oxygen to tumor cells. Owing to its dual mechanism of action, BMS-794833 represents a promising drug for the treatment of osteosarcoma. It attenuates the aggressive behavior of osteosarcoma by simultaneously inhibiting the VEGFR pathway, thereby mitigating the growth and metastatic potential of osteosarcoma. In addition, its inhibitory effects on MET offer additional therapeutic advantages. In this study, BMS-794833 and anlotinib were found to exert synergistic effects against osteosarcoma *in vitro* and *in vivo*. However, the survival and proliferation of osteosarcoma also rely on the bone microenvrionment, which formes the immunosuppressive microenvironment including the overactivated molecules and cells. Our animal model was based on the subcutaneous injection of osteosarcoma cells [43]. Therefore, the influences of bone microenvironment weren’t considerated in this model and warrants further investigation.

In conclusion, this study demonstrated that BMS-794833 attenuated anlotinib resistance in osteosarcoma by targeting the VEGFR/Ras/CDK2 pathway. In addition, BMS-794833 affected the resistance of osteosarcoma cells to anlotinib through the EMT and apoptosis pathways. Importantly, BMS-794833 and anlotinib exerted synergistic therapeutic effects against osteosarcoma *in vivo*. Altogether, this study reveals a novel VEGFR-targeting drug that can be used in combination with anlotinib for the treatment of osteosarcoma, providing a theoretical basis for addressing anlotinib resistance.

## CRediT authorship contribution statement

**Qingtao Meng:** Funding acquisition, Data curation, Conceptualization. **Jian Han:** Investigation, Data curation. **Peng Wang:** Writing – original draft, Formal analysis. **Chenxu Jia:** Writing – original draft. **Mingyang Guan:** Validation. **Bolun Zhang:** Methodology. **Wenzhi Zhao:** Writing – review & editing, Writing – original draft, Supervision.

## Declaration of competing interest

The authors declare that they have no known competing financial interests or personal relationships that could have appeared to influence the work reported in this paper.
