# Supplementary figures and images for: BMS-794833 reduces anlotinib resistance in osteosarcoma by targeting the VEGFR/Ras/CDK2 pathway
Source: J Bone Oncol. 2024 Mar 16;45:100594. doi: 10.1016/j.jbo.2024.100594 (PMC10963651; doi:10.1016/j.jbo.2024.100594)

Figure 6D

DMSO 20X


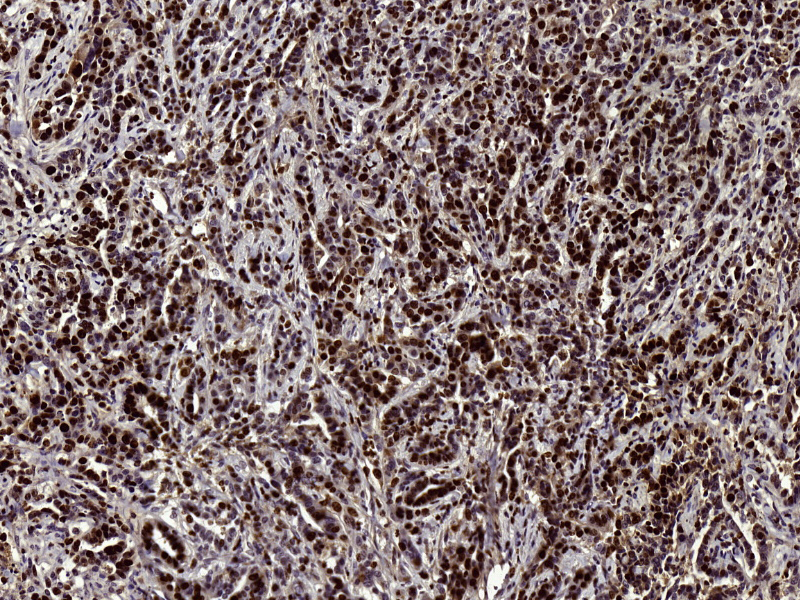


DMSO 200X


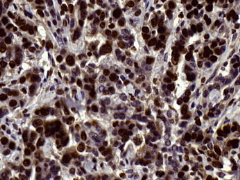


BMS-794833 20X


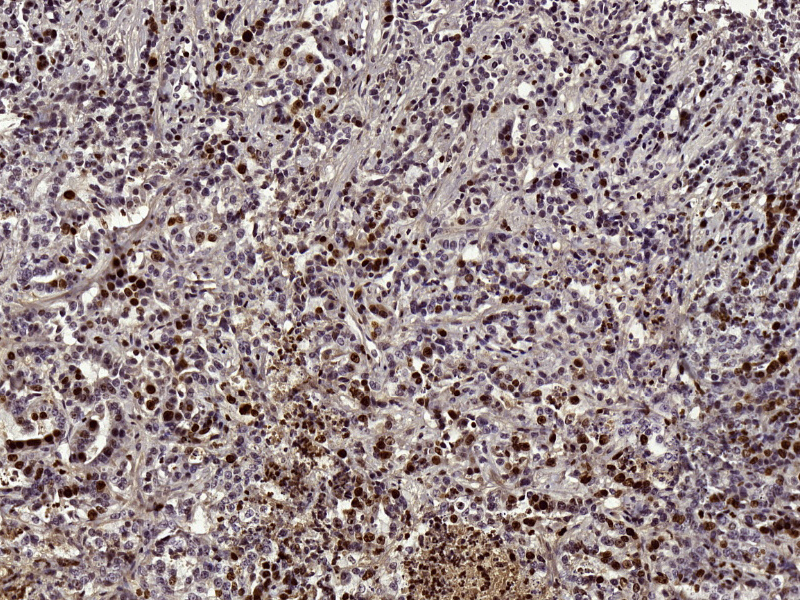


BMS-794833 200X


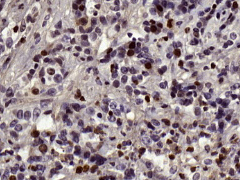


Anlotinib 20X


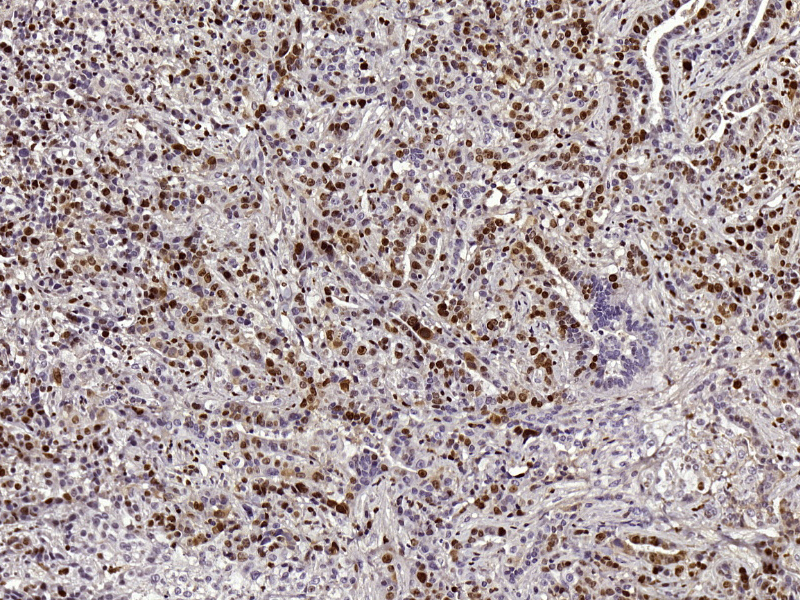


Anlotinib 200X


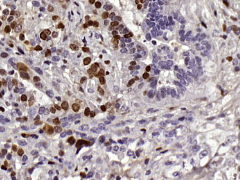


BMS-794833+Anlotinib 20X


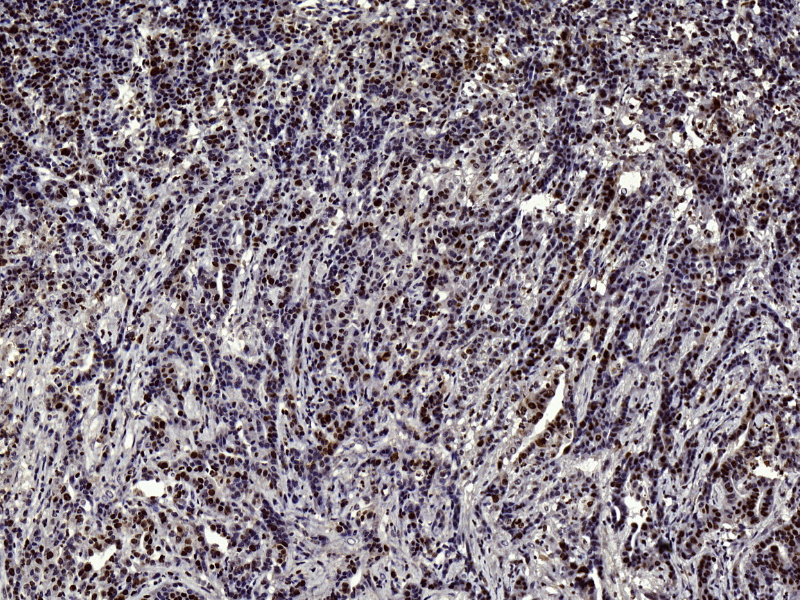


BMS-794833+Anlotinib 200X


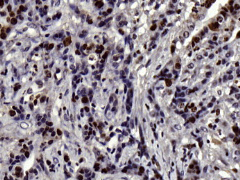

Supplement: Supplementary data 1 [file mmc1.docx]
