# Supplementary material for: BMS-794833 reduces anlotinib resistance in osteosarcoma by targeting the VEGFR/Ras/CDK2 pathway
Source: J Bone Oncol. 2024 Mar 16;45:100594. doi: 10.1016/j.jbo.2024.100594 (PMC10963651; doi:10.1016/j.jbo.2024.100594)
Supplement: Supplementary data 2 [file mmc2.doc]

Supplementary Figure 1


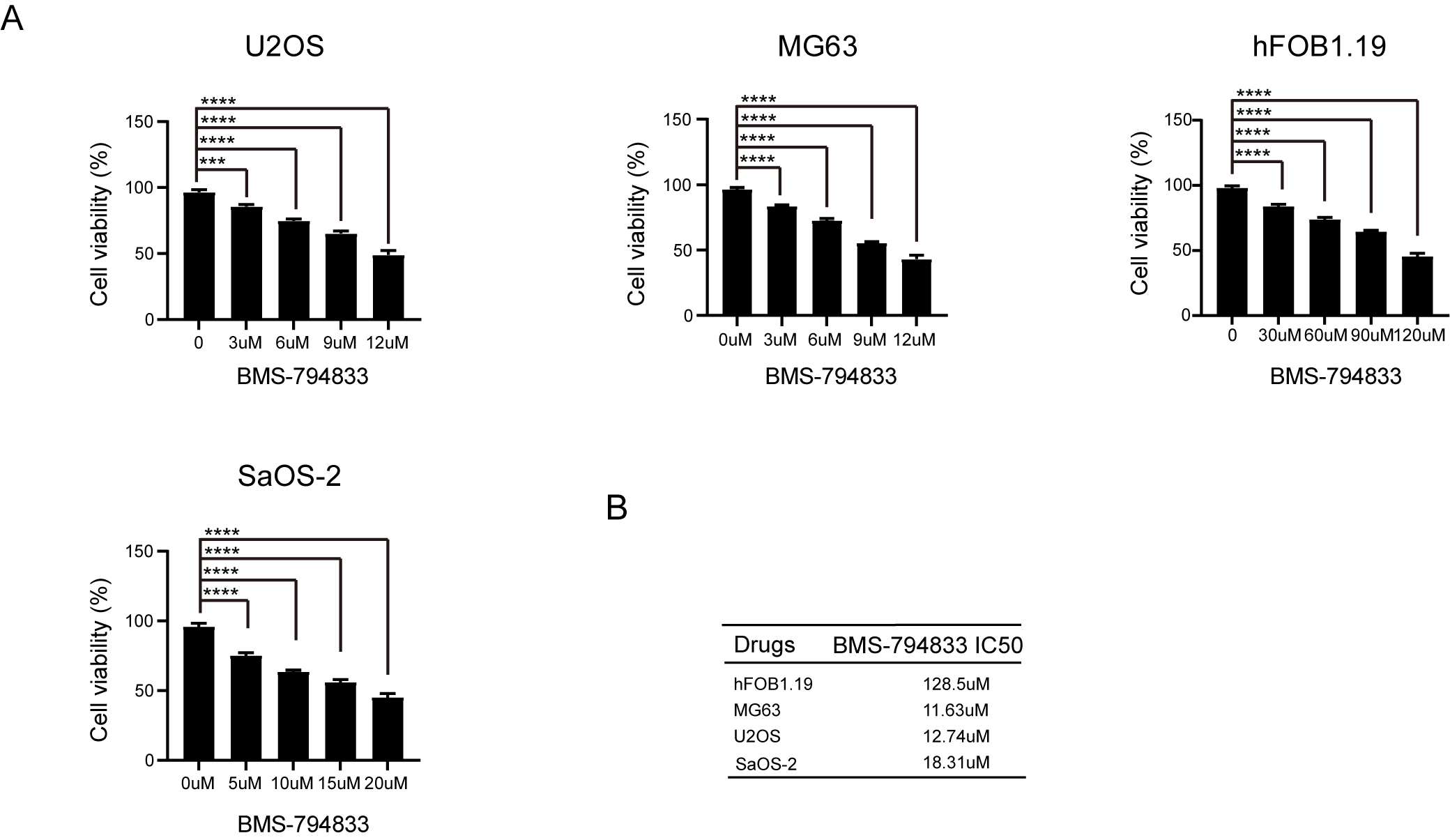


(A-B) The U2OS, MG63, hFOB1.19 and SaOS-2 cells were treated with BMS-794833 respectively, and the viability of the cells was detected by CCK8 assay, and the IC50 value was calculated.

Supplementary Figure 2


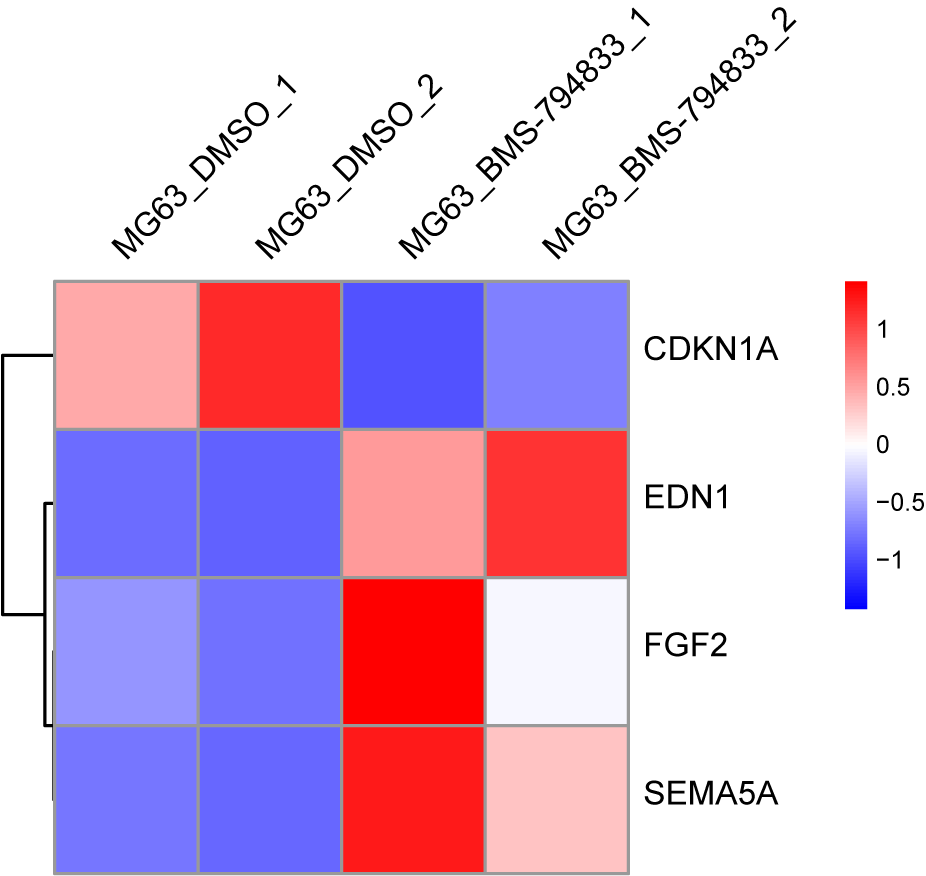


1. Heat map of proliferation-related genes treated with DMSO and BMS-794833.

Supplementary Figure 3


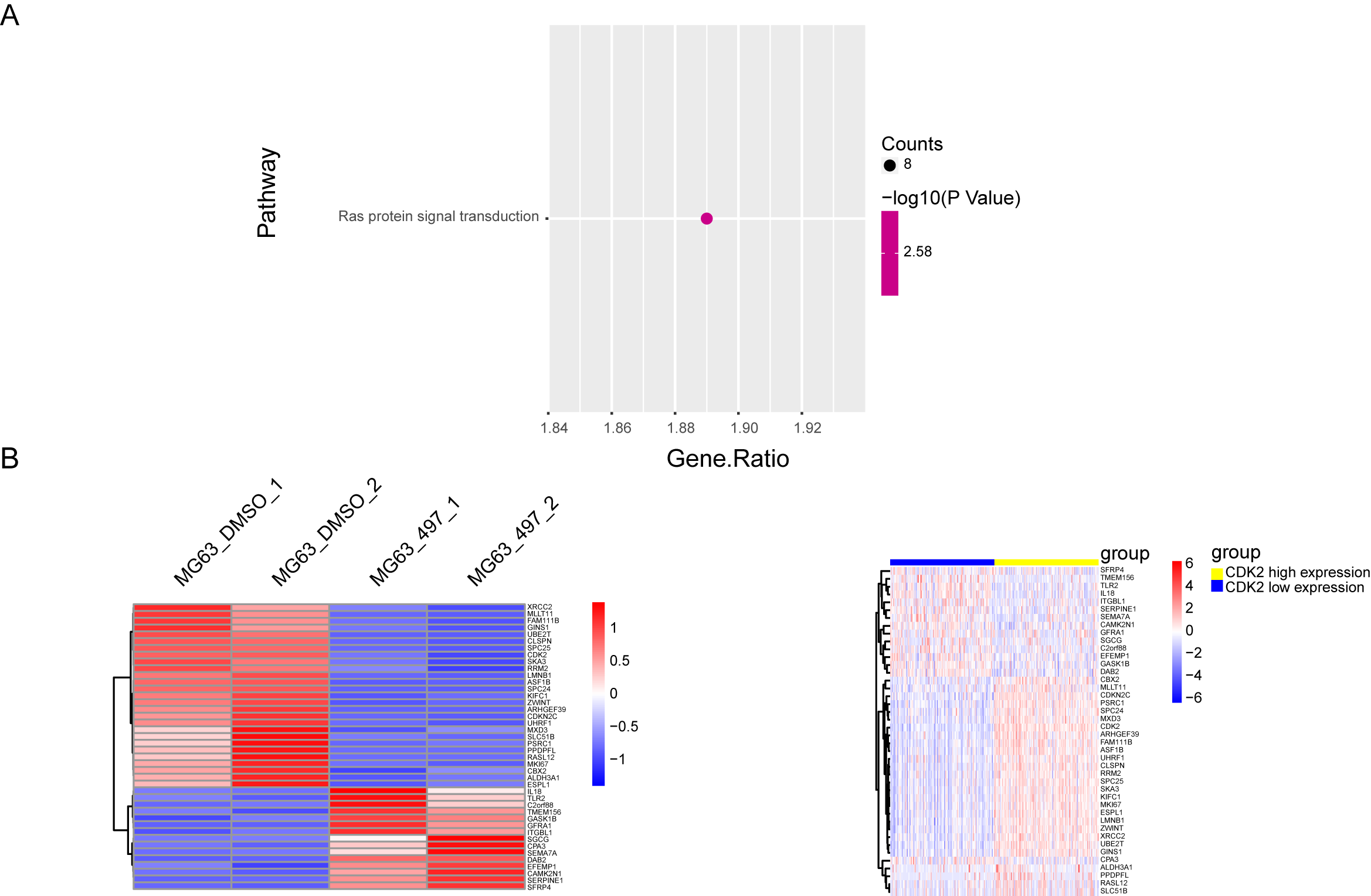


(A) The bubble map showed that differential genes were enriched in Ras pathway.

(B) Heat map showing differential genes with low and high CDK2 expression.

Supplementary Figure 4


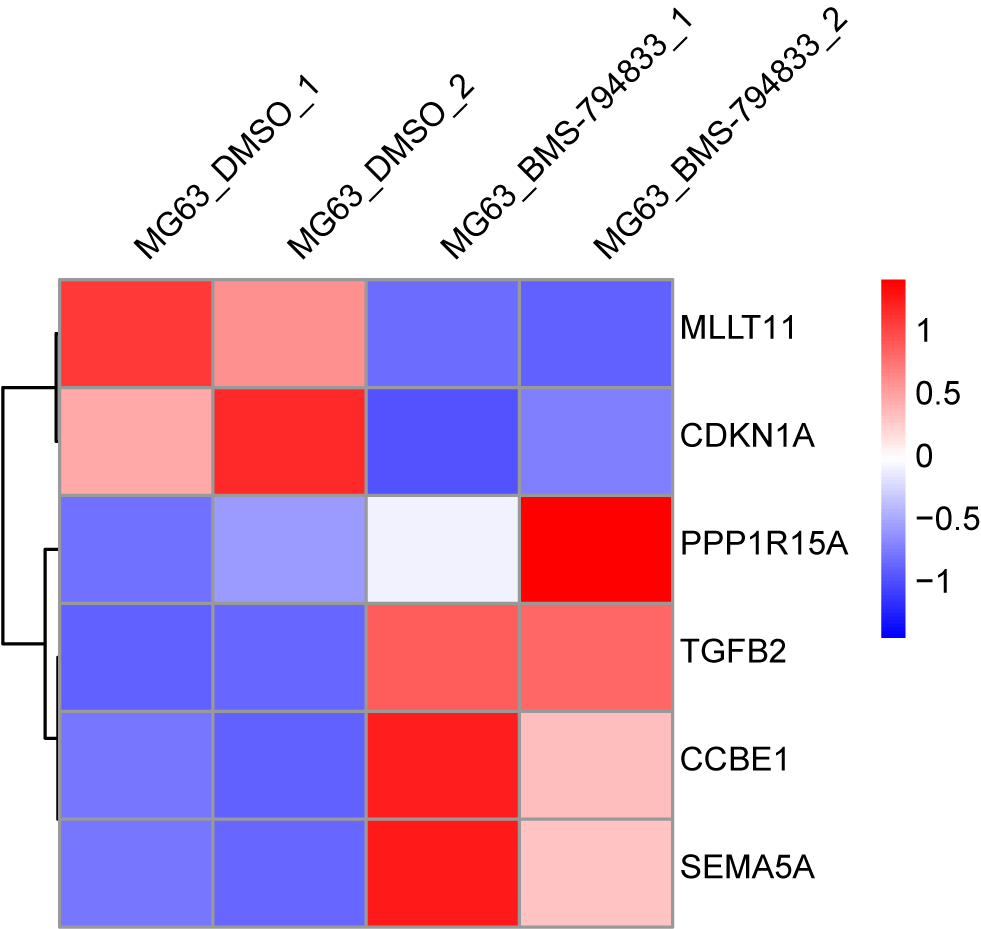


1. Heat maps of apoptosis and EMT-related genes after treatment with DMSO and BMS-794833.

Supplementary Figure 5


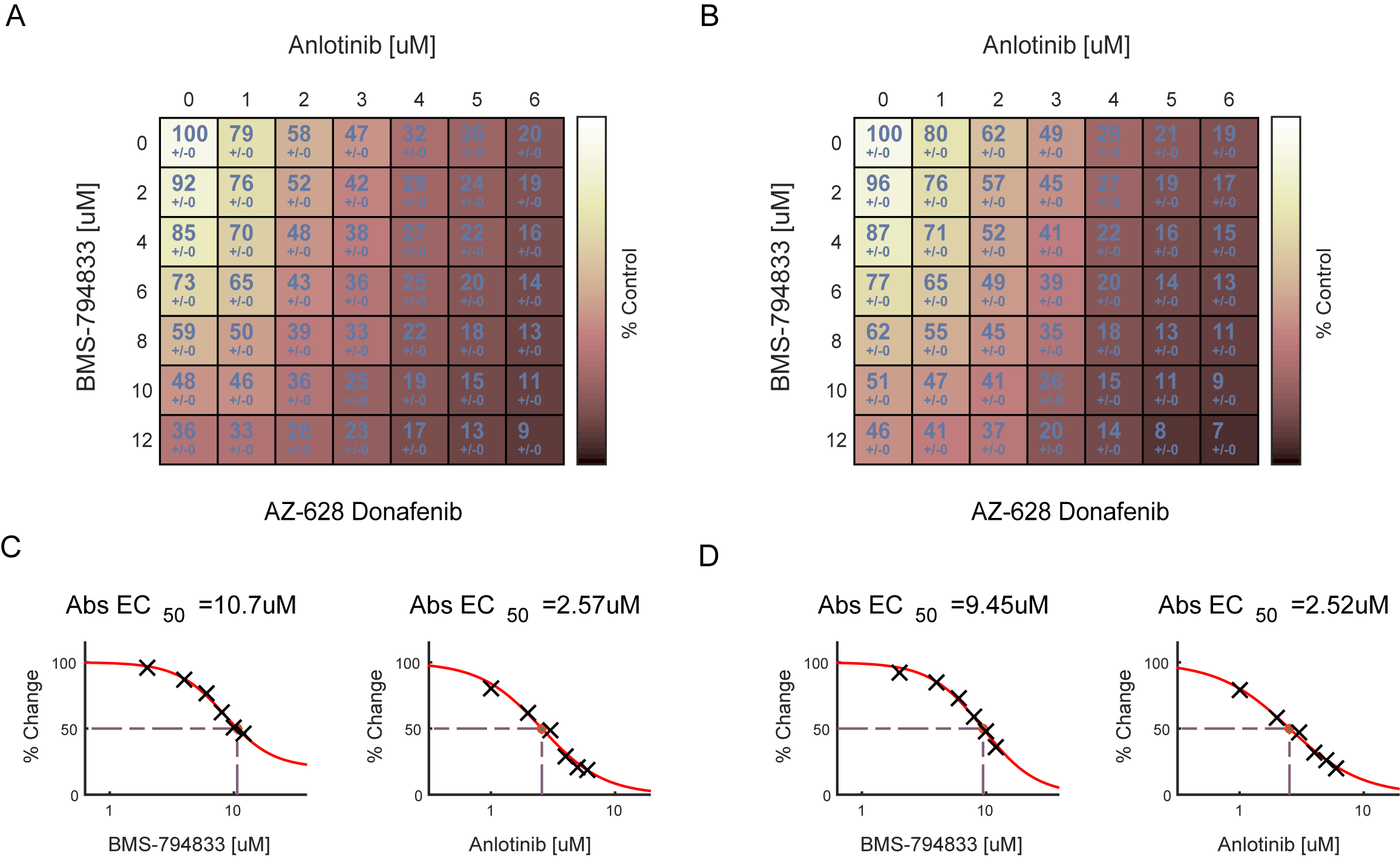


(A-D) 2D visualization of synergy between BMS-794833 and anlotinib at various concentrations in two different osteosarcoma cell cultures (24h treatment) by combenefit2.

Supplementary Figure 6


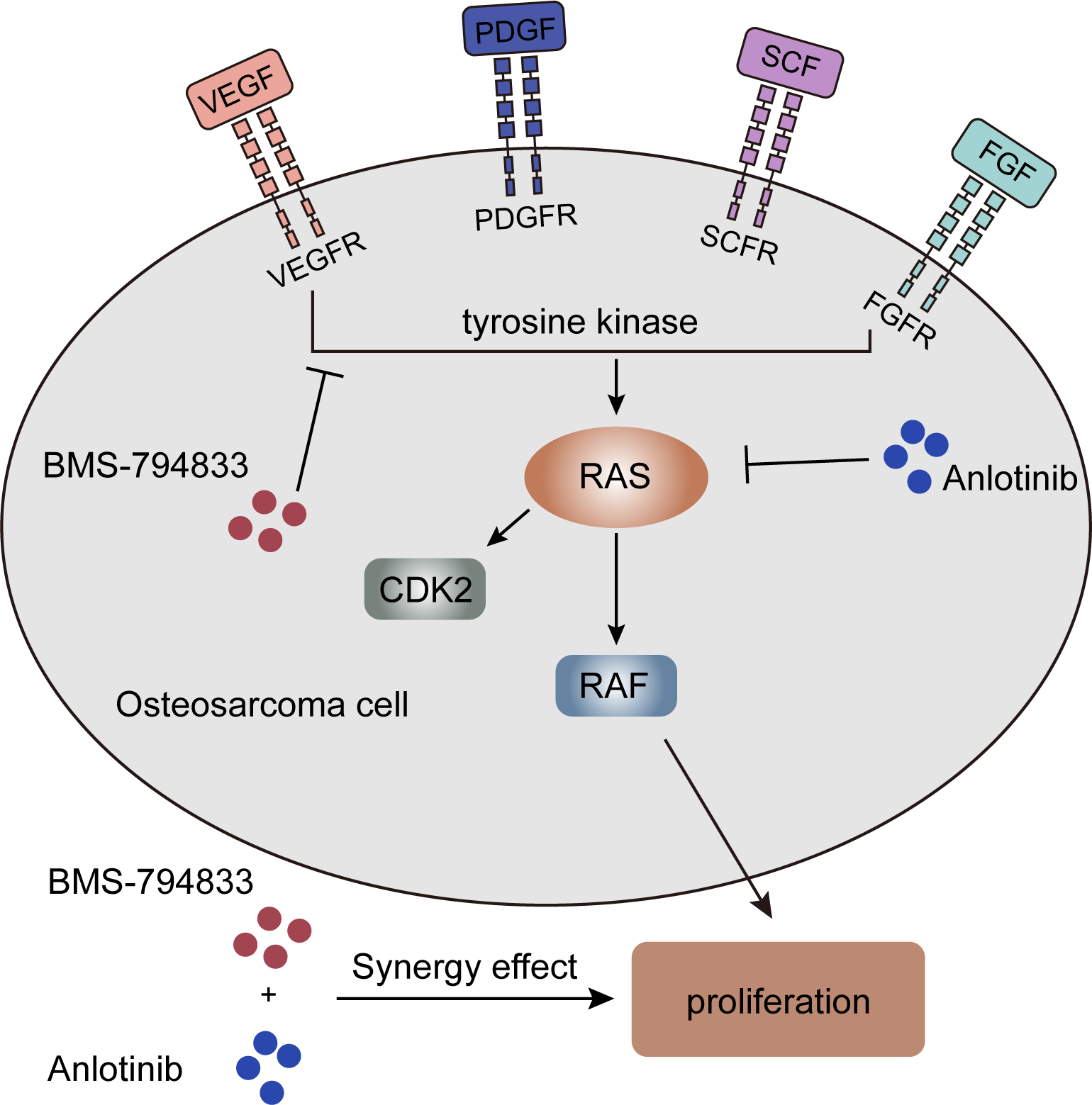


1. Diagram of the mechanism of this study.
